# Double Stimulation in the Same Ovarian Cycle (DuoStim) to Maximize the Number of Oocytes Retrieved From Poor Prognosis Patients: A Multicenter Experience and SWOT Analysis

**DOI:** 10.3389/fendo.2018.00317

**Published:** 2018-06-14

**Authors:** Alberto Vaiarelli, Danilo Cimadomo, Elisabetta Trabucco, Roberta Vallefuoco, Laura Buffo, Ludovica Dusi, Fabrizio Fiorini, Nicoletta Barnocchi, Francesco Maria Bulletti, Laura Rienzi, Filippo Maria Ubaldi

**Affiliations:** ^1^Clinica Valle Giulia, G.en.e.r.a. Centers for Reproductive Medicine, Rome, Italy; ^2^Clinica Ruesch, G.en.e.r.a. Centers for Reproductive Medicine, Naples, Italy; ^3^G.en.e.r.a. Veneto, G.en.e.r.a. Centers for Reproductive Medicine, Marostica, Italy; ^4^G.en.e.r.a. Umbria, G.en.e.r.a. Centers for Reproductive Medicine, Umbertide, Italy; ^5^University of Targu Mures, Targu Mures, Romania

**Keywords:** duostim, double stimulation, dual-stimulation, low prognosis patients, poor responder, IVF, euploid blastocyst, Poseidon

## Abstract

A panel of experts known as the POSEIDON group has recently redefined the spectrum of poor responder patients and introduced the concept of suboptimal response. Since an ideal management for these patients is still missing, they highlighted the importance of tailoring the ovarian stimulation based on the chance of each woman to obtain an euploid blastocyst. Interestingly, a novel pattern of follicle recruitment has been defined: multiple waves may arise during a single ovarian cycle. This evidence opened important clinical implications for the treatment of poor responders. For instance, double stimulation in the follicular (FPS) and luteal phase (LPS) of the same ovarian cycle (DuoStim) is an intriguing option to perform two oocyte retrievals in the shortest possible time. Here, we reported our 2-year experience of DuoStim application in four private IVF centers. To date, 310 poor prognosis patients completed a DuoStim protocol and underwent IVF with blastocyst-stage preimplantation-genetic-testing. LPS resulted into a higher mean number of oocytes collected than FPS; however, their competence (i.e., fertilization, blastocyst, euploidy rates, and clinical outcomes after euploid single-embryo-transfer) was comparable. Importantly, the rate of patients obtaining at least one euploid blastocyst increased from 42.3% (*n* = 131/310) after FPS to 65.5% (*n* = 203/310) with the contribution of LPS. A summary of the putative advantages and disadvantages of DuoStim was reported here through a Strengths–Weaknesses–Opportunities–Threats analysis. The strengths of this approach make it very promising. However, more studies are needed in the future to limit its weaknesses, shed light on its putative threats, and realize its opportunities.

## Introduction

In IVF, poor response to controlled ovarian stimulation (COS) represents an important issue, which may affect 9–24% of the infertile women (1). Such a wide range is indeed indicative of a heterogeneous population of patients. Hence, several definitions have been proposed to classify “poor responders,” namely up to 41 according to the systematic review by Polyzos and Devroey (2), and numerous protocols have been adopted to treat these women. The Bologna criteria (3) represented the first successful attempt to outline some guidelines in the definition of poor ovarian response. At least two of the following characteristics must be present to define “a poor responder patient”: advanced maternal age (>40 years) and/or scarce response to a previous conventional stimulation (≤3 oocytes) and/or reduced ovarian reserve (antral follicle count, AFC < 5–7 follicles, and/or AMH < 1.1 ng/ml).

Yet, some criticism arose, since oocyte competence may be severely affected from numerous factors, among which maternal age is the most important (4, 5), to point out that the classification should be more patient-oriented and match the putative number of retrievable oocytes with their putative chance to develop as an euploid blastocyst. Hard evidence support that both the number of retrieved oocytes and woman age are indeed the most important parameters to predict the chance to conceive after IVF for all patients, including poor prognosis ones (6–9). An efficient prediction of the ovarian response is, therefore, pivotal to define a tailored-COS for each patient, especially poor responders, which should be based upon AFC and AMH, namely, the most widely used biomarkers at present (10).

A new classification by a panel of experts, known as the POSEIDON (Patient-Oriented Strategies Encompassing IndividualizeD Oocyte Number) group (11), has been introduced to better categorize the spectrum of poor responder patients. Currently, the treatment for this heterogeneous group is not evidence-based, yet, and the prognosis is highly dependent upon patients’ specific characteristics, rather than upon the COS protocol chosen (9). The POSEIDON group highlighted instead the importance of tailoring the stimulation based on the chance of each woman to obtain an euploid blastocyst, proposed as novel main goal of COS. Indeed, blastocyst transfer (12), especially of euploid embryos (13), showed to date the most promising results per transfer achievable in IVF. The POSEIDON group then introduced the concept of “sub-optimal response.” In this group of patients collecting 4–9 oocytes, 4 sub-clusters were outlined according to both the ovarian reserve and the maternal age. Specifically, groups 3 and 4 are represented from women younger than 35 or older than 35, respectively, with a compromised ovarian reserve (AFC < 5 and AMH < 1.2 ng/ml), an issue, which cannot be resolved pharmacologically, as already reported in several studies (14–21).

The aim of this paper is to provide an update about the IVF management of poor responders, as well as to describe and encourage the use of novel strategies, especially for the patients of POSEIDON groups 3 and 4, to increase the cumulative live birth per IVF treatment.

## Theories of Follicle Recruitment

Follicular development is an extremely dynamic process. According to the classic theory (*single recruitment episode theory*), a single cohort of antral follicles grows during the follicular phase of the ovarian cycle after luteal regression. However, this theory has been overtaken by the evidence of multiple waves arising during an ovarian cycle in many mammals. Such evidence, at first reported in large animal models (22–27), was confirmed also in humans leading to the definition of two further theories of follicle recruitment (28): *the continuous recruitment theory*, according to which the follicles start growing and regress continuously during the ovarian cycle; and *the waves theory*, according to which 2–3 cohorts of antral follicles are recruited per ovarian cycle. However, the mechanisms underlying follicular recruitment have not been fully elucidated yet. Several intraovarian regulators, FSH and progesterone levels, inflammatory markers (e.g., serum C reactive protein) were all proposed as modulators of the dynamics behind the origin of follicular waves (28–30). From a clinical perspective, the growing knowledge of human ovarian follicular waves, opened new options for COS to improve the efficiency and possibly the efficacy of IVF.

## DuoStim: Considerations, Indications, and Framework

Currently, there is insufficient evidence to recommend an ideal management of poor responders as defined through the Bologna Criteria. Indeed, regardless the COS protocol adopted, consistently low live birth rates were achieved in this population of patients (31–33). The choice of COS for patients with poor ovarian reserve markers and/or of advanced maternal age can be challenging. Yet, the number as well as the quality of the oocytes retrieved are important factors to increase the cumulative live birth rate. Moreover, these women have a limited time left to attempt to conceive with their own eggs: their “follicular heritage” suffers from a dramatic physiological decline of oocyte quantity and quality. The gonadotrophins can only support the growth of cohorts of follicles already present in the ovaries, but they cannot induce the *de novo* production of follicles. Therefore, increasing the dose of gonadotrophins administered or even adopting more powerful drugs will never compensate a reduced ovarian reserve.

In this scenario, a novel COS strategy has been proposed: double stimulation in the same ovarian cycle (DuoStim). Such protocol particularly suits poor prognosis and oncological patients, who require maximizing the exploitation of their ovarian reserve in a limited time (34–36). DuoStim, by combining conventional follicular phase stimulation (FPS) with luteal phase stimulation (LPS), can be considered a valuable option in patients with reduced ovarian reserve and/or advanced maternal age to maximize the number of oocytes retrieved in a single ovarian cycle, and for patients who did not collect oocytes or did not produce competent embryos after conventional FPS (37).

The very first experience with double stimulation has been reported by Kuang and colleagues (36) who showed that COS conducted in both the FPS and LPS of the same ovarian cycle results in the collection of oocytes with similar developmental competence (36). The drugs used for COS in the Shanghai protocol, as it was called in the paper, were clomiphene citrate 25 mg/day, letrozole 2.5 mg/day, and mild dose of human menopausal gonadotrophin 150–225 IU/day. Moreover, the final oocytes maturation was induced with triptorelin followed by ibuprofen 0.6 g the day of trigger and the day after, in both FPS and LPS. In 2016, we published our proof-of-concept study where a DuoStim protocol was adopted together with a pre-implantation genetic testing (PGT-A) program in poor prognosis patients (34). The most important outcome outlined by this study was that the application of DuoStim in this thorny patient population increased the chance of obtaining at least one euploid blastocyst in a single ovarian cycle from 40 to 70%. Contrary to the Shanghai protocol, the DuoStim protocol consists in a co-treatment with maximal dose of FSH plus LH and GnRh antagonist to prevent ovulation in both FPS and LPS. The rationale of administrating FSH 300 IU/day plus LH 75 IU/day in an antagonist protocol, instead of adopting a mild stimulation, is to limit the risk for cycle cancelation and possibly decrease time-to-pregnancy by maximizing the number of oocytes collected per stimulation. To this regard, mild stimulation has been associated with a reduced number of oocytes retrievable per COS cycle (38). Therefore, even if no randomized controlled trial (RCT) has been performed to compare mild versus conventional COS in a DuoStim protocol, it is reasonable to hypothesize that while the cost of the former COS approach might involve lower expense than the latter (39), effectiveness is questionable. This is especially true if we account cumulative live birth rate per started cycle as the measure of success in IVF (40, 41).

The patient drop-out is then another very important issue in the treatment of poor prognosis patients. It has been reported largely variable (20–60%) among couples undergoing IVF worldwide (42–44). Still, a generally valid information cannot be produced due to heterogeneity in terms of cost, reimbursement policies, accessibility to IVF, indication for PGT-A, etc., among the different countries (45, 46). Importantly, the most significant drop-out rate involves the second attempt after a first failed IVF cycle. Furthermore, when a second attempt is performed, ~10 months often pass from the former retrieval, while the time is crucial especially for poor prognosis patients (47). These cases might be rescued *via* the application of a DuoStim approach, which would at least allow to conduct two retrievals in a single ovarian cycle. A future RCT comparing double FPS versus DuoStim and entailing also the drop-out rate among the outcomes under investigation might provide an answer to this issue.

### Indications to Duostim

Since October 2015, DuoStim has been proposed at our four centers, after extensive counseling, to all patients matching at least two of the following criteria: AMH < 1.5 ng/mg, AFC ≤ 6 follicles, ≤5 metaphase II (MII) oocytes retrieved in a previous cycle, advanced maternal age (≥35 years). Importantly, a single parameter is insufficient to outline an indication to DuoStim, since AFC evaluation *per se* might be limited from large inter-operator variability and AMH measurement *per se* might be affected from sample handling, storage, and low inter-laboratory reproducibility (48–50).

Another possible application of this strategy is urgent fertility preservation, in case few mature oocytes are collected after conventional COS and the time left before starting cancer therapy allows it (51).

### Framework of a Duostim Protocol

To all patients undergoing Duostim, luteal estradiol priming (4 mg/day of estradiol valerate) was started in day 21 of the previous menstrual cycle to promote the synchronization and coordination of follicular growth (52, 53). After the transvaginal ultrasound and basal assessment of the ovaries, on day 2 to day 3 of the menstrual cycle, luteal estradiol priming was stopped, and FPS was started with fixed dose of rec-FSH 300 IU/day plus LH 75 IU/day for 4 days. Follicular growth was monitored on day 5 and then every 2–3 days. GnRh antagonist was administered daily after the identification of a leading follicle with a diameter ≥ 13–14 mm in FPS and LPS until the day of ovulation trigger. The final maturation of oocytes was triggered by a subcutaneous bolus of buserelin (dose 0.5 ml) to reduce the time of luteolysis. Egg retrieval was performed 35 h after the trigger. ICSI, blastocyst culture, trophectoderm biopsy, and vitrification, were performed as described in detail elsewhere (8, 54–56). Five days after the first retrieval, namely, the time needed to complete luteolysis (57), LPS was started with the same protocol and daily dose regardless of the number of antral follicles visible through ultrasound scan in the anovulatory wave. A freeze-all approach was adopted and the biopsy fragments from both stimulations were shipped together and analyzed in the same run at an external genetic lab (Igenomix, Italy). In presence of euploid blastocyst(s), frozen single embryo transfers were performed in a modified-natural or artificial cycle (58).

## Multicenter Experience at G.EN.E.R.A. Centers for Reproductive Medicine (Rome, Naples, Umbertide, and Marostica, Italy) to Date

DuoStim was suggested to 353 consecutive couples approaching G.EN.E.R.A. centers for reproductive medicine (Rome, Naples, Marostica, and Umbertide, Italy) between October 2015 and December 2017. All the related data were prospectively recorded in a relational database [Fertilab Manager (FLM), Italy]. Among them, 17 did not respond to FPS and were excluded from this analysis (4.8%). Then, 336 patients underwent LPS and 26 (7.7%) did not respond. The 43 patients who did not respond to either FPS or LPS were stopped after 8–9 days of gonadotrophins administration. Overall, 310 patients completed the DuoStim approach with two oocyte retrievals of at least one cumulus-oocyte-complex in a single menstrual cycle and were included in this analysis (Figure S1A in Supplementary Material). The maternal age of the patients included in the analysis was 40.0 ± 3.0 years (33.0–44.0), the AFC was 5.3 ± 2.5 (3–13), the AMH was 1.0 ± 1.0 (0.1–2), and they already underwent 1.0 ± 1.3 (0–6) previous IVF cycles collecting 4.0 ± 2.6 (0–14) MII oocytes. LPS was on average 1 day longer than FPS. No increase of the post-oocyte retrieval complications has been reported so far compared to FPS-only cycles.

**Figure 1:**
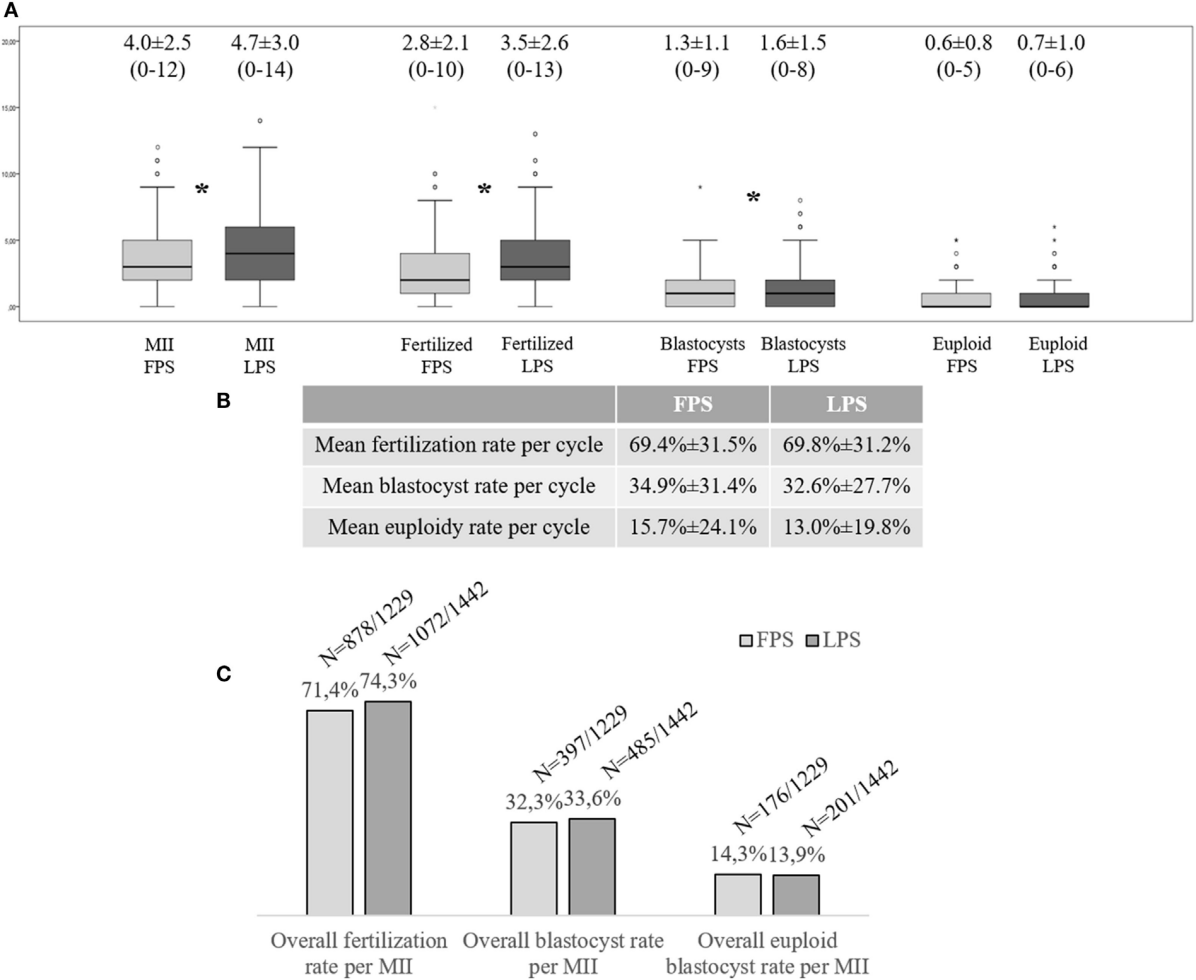
Multicenter clinical experience at the G.EN.E.R.A. centers for reproductive medicine (Rome, Naples, Marostica, and Umbertide) with the application of a DuoStim approach. **(A)** Mean number of metaphase (MII) oocytes, fertilized embryos, blastocysts, and euploid blastocysts obtained per cycle after follicular phase stimulation (FPS) and luteal phase one (LPS); **(B)** Mean embryological results calculated per MII oocyte retrieved and inseminated in FPS- and LPS-derived cycles; **(C)** Overall embryological results of the MII oocytes collected after FPS and LPS, respectively. The stars identify statistically significant differences. The non-Gaussian distribution of the data was assessed through the Shapiro–Wilk test. Wilcoxon signed-rank test and Fisher’s exact test were used to test for significant differences between FPS- and LPS-derived data.

299 FPS- (96.5%) and 298 LPS-derived (96.1%) oocyte retrievals resulted in at least one MII oocyte collected (Figure S1B in Supplementary Material). Figure 1A displays the number of MII oocytes, fertilized oocytes, and blastocysts obtained on average after each LPS and FPS, respectively. Interestingly, a higher number of oocytes was collected after LPS, which involved also a higher number of fertilized oocytes and blastocysts (Wilcoxon Signed Rank test: *p* < 0.01). No difference was reported to date in terms of mean number of euploid blastocysts obtained from this cohort. The mean fertilization, blastocyst, and euploid blastocyst rates calculated per number of MII oocytes collected from each cycle (FPS- and LPS-derived ones, respectively) are reported in Figure 1B and were similar in the two groups. The overall fertilization, blastocyst, and euploid blastocyst rates of the 1,229 and 1,442 MII oocytes obtained after FPS and LPS, respectively, were also similar (Figure 1C).

229 (73.9%) and 230 (74.2%) patients obtained at least one blastocyst after FPS and LPS, respectively. This resulted in 280 patients who obtained at least one blastocyst in a single menstrual cycle due to DuoStim (90.3%). 131 (42.3%) and 129 (41.6%) patients obtained at least one euploid blastocyst after FPS and LPS, respectively. This resulted in 203 (65.5%) patients who obtained at least one euploid blastocyst in a single menstrual cycle due to DuoStim (Figure S1B in Supplementary Material).

81 and 83 FPS-derived and LPS-derived single euploid blastocyst transfers have been performed, respectively. In presence of euploid blastocysts from both FPS and LPS, the embryo to transfer was randomly chosen. The positive pregnancy rates were 48.1% (*n* = 39/81) and 59.0% (*n* = 49/83; Fisher’s exact test: *p* = NS). The biochemical pregnancy loss rates were 7.7% (*n* = 3/39) and 8.2% (*n* = 4/49; *p* = NS). The miscarriage rates were 11.1% (*n* = 4/36) and 8.9% (*n* = 4/45; *p* = NS). Therefore, the ongoing pregnancy rates were 39.5% (*n* = 32/81) and 49.4% (*n* = 41/83; *p* = NS) (Table S1 in Supplementary Material).

## Discussion

This perspective paper dealing with the definition and implementation of DuoStim highlights the value of this strategy in treating poor prognosis patients. Importantly, the competence of the oocytes collected after both stimulations conducted in the FP and LP is similar in terms of fertilization, blastulation, and euploidy rates, as well as clinical outcomes after single euploid blastocyst transfer. However, the LPS seems to induce a better exploitation of the ovarian reserve with almost one more oocyte on average collected with respect to the FPS. Interestingly, these data further support the exploitation of anovulatory waves of follicle recruitment to obtain competent oocytes (34, 59–64). This practice is in countertendency with respect to the ovarian physiological behavior, but apparently it may be very successful. However, more stimulation cycles were canceled in the LP due to no response to the stimulation with respect to the FP.

The idea of DuoStim has been initially proposed to manage patients with poor ovarian reserve. However, the POSEIDON group highlighted the importance of obtaining at least one euploid embryo after COS as novel primary outcome in IVF. Therefore, based on this new concept, DuoStim in the future could be proposed not only *a priori* according to the inclusion criteria previously defined in this paper but also *post hoc* according to the number of blastocysts obtained after FPS. Clearly, the decision to perform also LPS (i.e., DuoStim) should depend on the expected euploidy rate of those FPS-derived blastocysts. To this end, the combination between the maternal age at oocyte retrieval and the number of embryos obtained after FPS represent the most predictive scheme to make a more appropriate choice (6, 7). Instead, in case of unexpectedly positive outcomes after FPS only (i.e., higher blastocyst rate than expected), we can consider avoiding LPS. Future studies should be properly designed to validate this putative strategy.

The data reported in this paper represent a further evidence to support the use of DuoStim to increase the number of poor prognosis patients obtaining an euploid blastocyst in a single menstrual cycle. No embryological, gynecological, or clinical issue has been reported to date. Yet, more biological, obstetrical, and neonatal evidence of safety is required, as well as an analysis of its cost-effectiveness.

### SWOT Analysis of DuoStim

**Figure 2:**
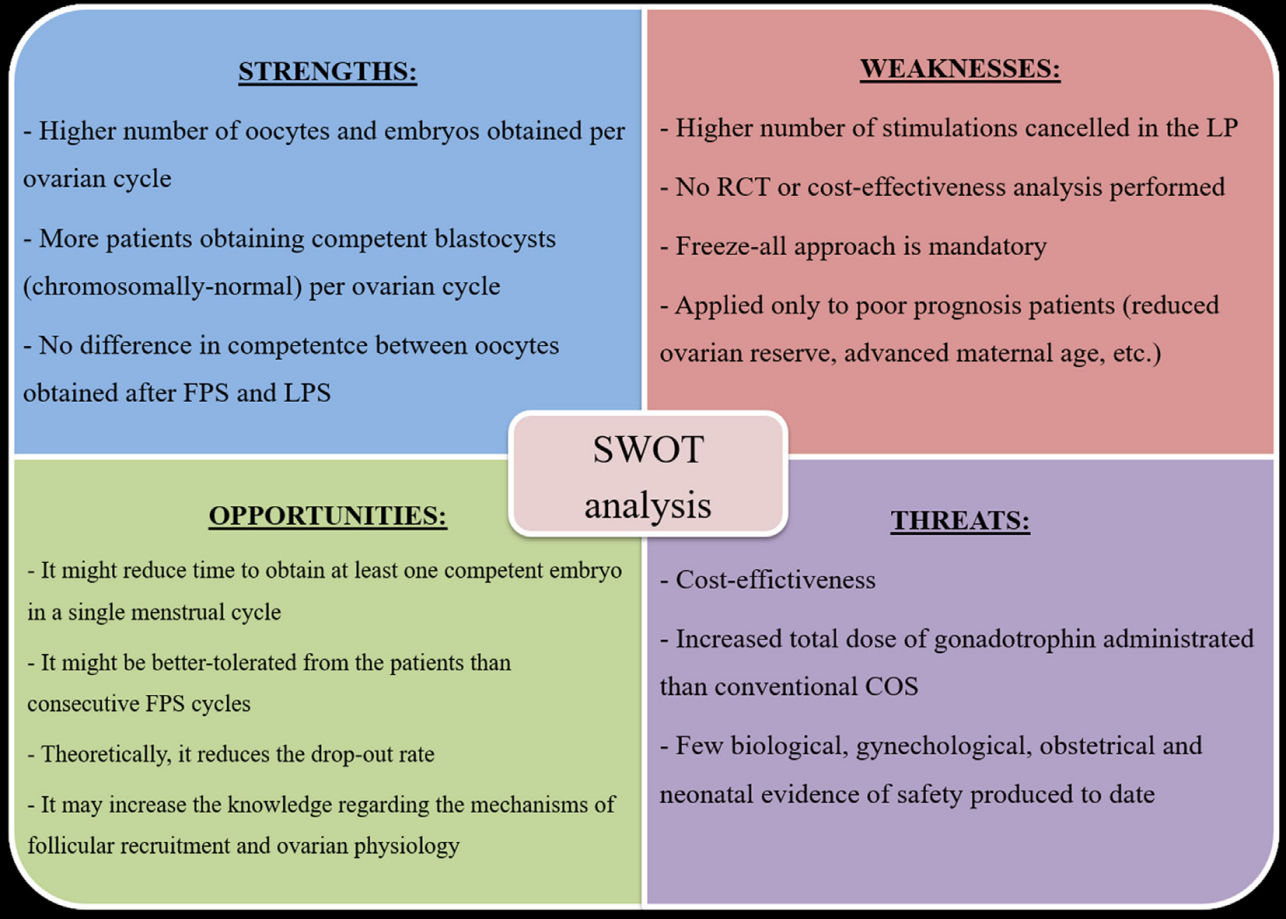
DuoStim SWOT analysis. Abbreviations: FPS, follicular phase stimulation; LPS, luteal phase stimulation; RCT, randomized controlled trial; COS, controlled ovarian stimulation.

To summarize the putative advantages and disadvantages of DuoStim, we conducted a SWOT analysis (Figure 2), namely an efficient analytical framework useful to summarize the *Strengths, Weaknesses, Opportunities*, and *Threats* of a technology. The strengths are: a higher number of oocyte (and embryos) might be obtained per ovarian cycle; more patients obtaining a (chromosomally normal) blastocyst per ovarian cycle; no difference has been reported to date in terms of competence between oocytes obtained after FPS and LPS. The weaknesses are: a higher number of stimulations seems to be canceled in the LP than in the FP; no RCT or cost-effectiveness analysis has been performed to date investigating the use of DuoStim; a freeze-all approach is mandatory; it has been applied only to poor prognosis patients. The opportunities are: a decrease in the time and increase in the chance to obtain at least one competent embryo in a single menstrual cycle; the DuoStim protocol might be better-tolerated from the patients than consecutive FPS cycles; the drop-out rate might be reduced; the knowledge regarding the mechanisms of follicular recruitment and ovarian physiology might be increased. The threats are: an analysis of the cost-effectiveness is yet eagerly needed; the total dose of gonadotrophins to be administrated is substantial; few biological, gynecological, obstetrical, and neonatal evidence of safety have been produced to date. The strengths of this approach make it very promising. However, more studies are needed in the future to limit its weaknesses, shed light on its putative threats, and realize its opportunities.

## Conclusion

The evidence that multiple waves of follicle recruitment may arise during a single ovarian cycle in women opened important clinical implications for the treatment of poor prognosis patients. LPS in general has become a promising protocol for patients who need to collect the highest number of oocytes in the shortest possible time (e.g., oncological patients). DuoStim approach conjugates FPS to LPS with very successful results reported to date. Still, any stimulation protocol, which exploits anovulatory waves of follicle recruitment should undergo a thorough biological and clinical investigation before it can be generally implemented. To this regard, DuoStim still needs a more extensive and wider validation to testify its safety. Interesting future perspectives to investigate its clinical efficacy/efficiency would entail (i) a RCT comparing double-FPS versus DuoStim; (ii) the application of DuoStim in cancer patients for fertility preservation; (iii) as well as in prospective analyses focused on patients clustered according to either the Bologna criteria or the Poseidon stratification. Until such evidence would be produced, DuoStim should be clinically applied only to a population of patients of poor prognosis and/or to whom time represents a critical issue.

## Ethics Statement

The institutional review board of the involved clinics approved this study. The project was reviewed by two different members of each committee, none directly involved in the study to exclude any potential conflict of interest. All members approved the study. The study was performed in accordance with the local regulation and all patients gave written informed consent to it in accordance with the Declaration of Helsinki. This study was considered in line with the clinics’ protocols and standard procedures.

## Author Contributions

AV and DC analyzed the data and drafted the manuscript. All authors contributed to the interpretation and discussion of the data.

## Conflict of Interest Statement

The authors declare that the research was conducted in the absence of any commercial or financial relationships that could be construed as a potential conflict of interest.

## Supplementary Material

Figure S1 **(A)** Flowchart and **(B)** cycle outcomes of 2-year multicenter application of DuoStim at G.EN.E.R.A. centers for reproductive medicine (Rome, Naples, Marostica, and Umbertide). FPS, follicular phase stimulation; LPS, luteal phase stimulation; MII, metaphase II oocyte.
