# Supplementary figures and images for: Double Stimulation in the Same Ovarian Cycle (DuoStim) to Maximize the Number of Oocytes Retrieved From Poor Prognosis Patients: A Multicenter Experience and SWOT Analysis
Source: Front Endocrinol (Lausanne). 2018 Jun 14;9:317. doi: 10.3389/fendo.2018.00317 (PMC6010525; doi:10.3389/fendo.2018.00317)

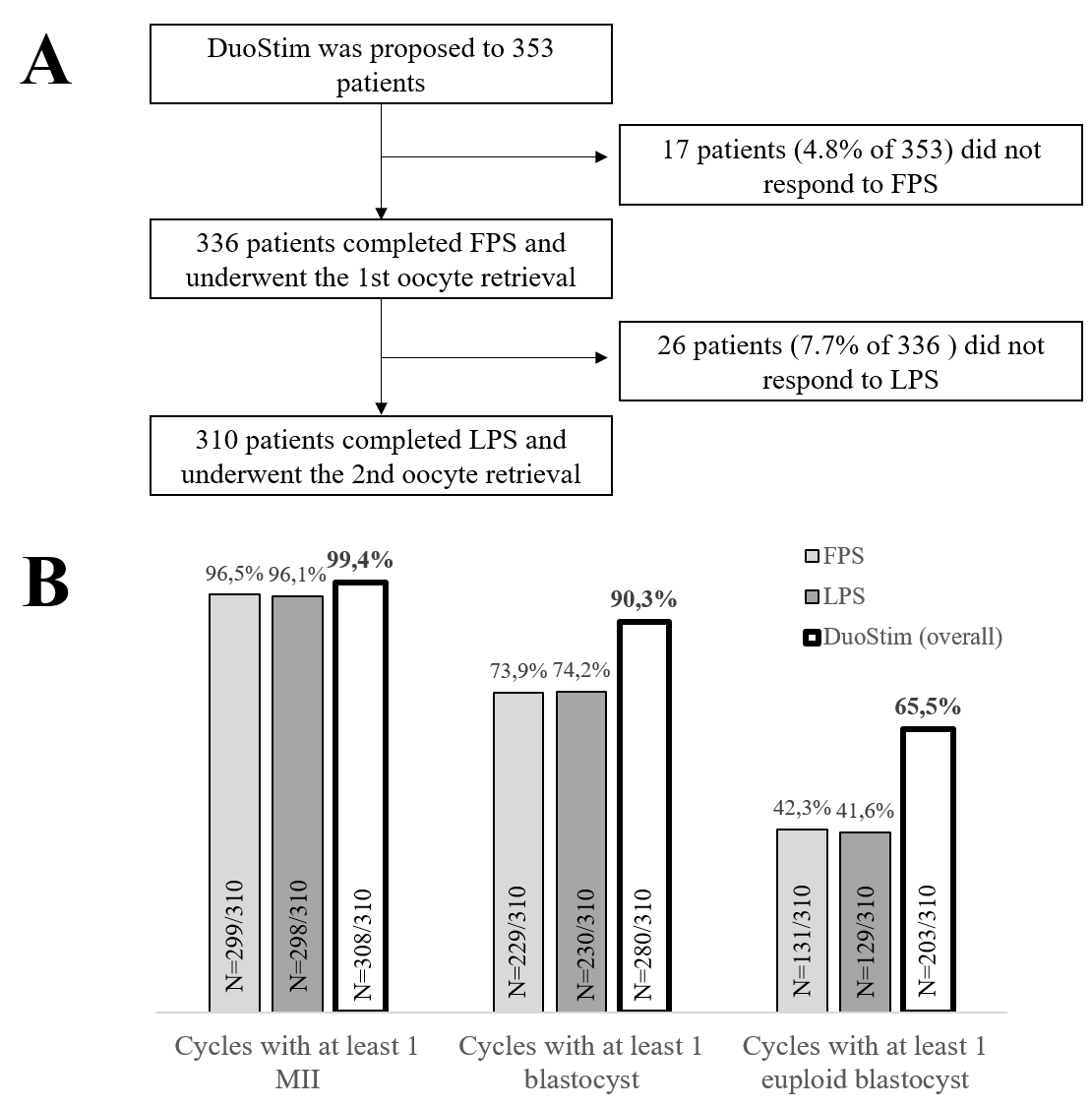

Supplement: Figure S1 — (A) Flowchart and (B) cycle outcomes of 2-year multicenter application of DuoStim at G.EN.E.R.A. centers for reproductive medicine (Rome, Naples, Marostica, and Umbertide). FPS, follicular phase stimulation; LPS, luteal phase stimulation; MII, metaphase II oocyte. [file image_1.tif]
