# Supplementary material for: Double Stimulation in the Same Ovarian Cycle (DuoStim) to Maximize the Number of Oocytes Retrieved From Poor Prognosis Patients: A Multicenter Experience and SWOT Analysis
Source: Front Endocrinol (Lausanne). 2018 Jun 14;9:317. doi: 10.3389/fendo.2018.00317 (PMC6010525; doi:10.3389/fendo.2018.00317)
Supplement: Supplementary file 2 [file table_1.docx]

**Supplementary Table 1. Preliminary clinical outcomes of euploid single blastocyst transfers of embryos obtained after follicular (FPS) or luteal phase stimulation (LPS).**

|  | **FPS** | **LPS** |
| --- | --- | --- |
| Number of euploid single blastocyst transfers | 81 | 83 |
| Number of positive pregnancy tests,  % of single blastocyst transfers (95%CI) | 39, *48.1% (37.0 to 59.5)* | 49, *59.0% (47.7 to 69.5)* |
| Number of biochemical pregnancy loss rates,  % of positive pregnancy tests (95%CI) | 3, *7.7% (2.0 to 22.0)* | 4, *8.2% (2.6 to 20.5)* |
| Number of clinical pregnancies | 36 | 45 |
| Number of miscarriages,  % of clinical pregnancies (95%CI) | 4, *11.1% (3.6 to 27)* | 4, *8.9% (2.9 to 22.1)* |
| Number of ongoing pregnancies(>22weeks)/deliveries,  % of single blastocyst transfers (95%CI) | 32, *39.5% (29.0 to 51.0)* | 41, *49.4% (38.3 to 60.5)* |
